# Social Cognitive Determinants of Dietary Behavior Change in University Employes

**DOI:** 10.3389/fpubh.2014.00023

**Published:** 2014-04-02

**Authors:** Shawna E. Doerksen, Edward McAuley

**Affiliations:** ^1^Department of Kinesiology and Community Health, University of Illinois at Urbana-Champaign, Champaign, IL, USA

**Keywords:** nutrition, social cognitive theory, self-efficacy, workplace, behavior

## Abstract

Many adults have poor dietary habits and few studies have focused on mechanisms underlying these behaviors. This study examined psychosocial determinants of dietary behavior change in university employes across a 5-month period. Participants completed measures of fruit and vegetable consumption (FVC) and low fat food consumption (LFC) and social cognitive constructs. Multiple regression analyses accounted for a unique proportion of variation in dietary change. Outcome expectations significantly predicted FVC and LFC. Self-efficacy significantly predicted LFC. Goals were not associated with dietary behaviors. Further research into implementation strategies may provide insight into how goals work to bring about change.

## Introduction

Currently, approximately one-third of the US adult population is obese (1). Obesity-related medical care is projected to add $48–66 billion in health care costs per year by 2030 (2). With such a dramatic increase in costs, the causes of obesity require attention. It has been reported that increase in energy intake accounts for much of the resultant obesity in the US (3). Dietary behaviors are important factors in the obesity crisis, in addition a host of other health benefits (4–9). Unfortunately, many Americans fail to meet the recommended guidelines for dietary behaviors (10) with less than a quarter of the US adult population consuming five or more servings of fruits and vegetables per day (11).

To effectively promote healthy nutrition in the remaining three quarters of the adult population, we must better understand the determinants of their behaviors and change in these behaviors. One important theory of behavior change is Bandura’s Social cognitive theory (SCT) (12, 13), which has been used extensively in the health behavior literature to predict health behaviors and elicit behavior change (14–16). Core constructs of this framework include self-efficacy, outcome expectations, self-regulation, and perceived impediments and facilitators of behavior (17) and they have been found to explain health behaviors, including dietary intake. Self-efficacy is fundamental to the process of behavior change in that confidence in ones’ abilities can provide the motivation necessary to follow through with a change in behavior (17). Additionally, self-efficacy is important because it influences several other SCT variables (17). Individuals who are more efficacious are more likely to believe that the behavior will bring about positive consequences. That is, they have positive outcome expectations regarding the behavior of interest. If an individual has higher self-efficacy, they believe that with personal effort, they can overcome the barriers to certain behaviors. Those who have high self-efficacy also set their personal goals higher than those who have lower levels of self-efficacy and are more motivated to achieve these goals (17). Not only does self-efficacy directly influence behavior, but also it has an indirect influence on a behavior by way of other social cognitive variables. Using these constructs together allows for a more comprehensive examination of behaviors and the potential to effectively target health behavior change.

Anderson et al. (18) found that these SCT constructs (as well as some demographic variables) accounted for 35% of the variance in fat intake, and 61% of the variance in fruit and vegetable consumption (FVC) in community-dwelling adults. Such findings provide initial cross-sectional evidence for the potential importance of social cognitive constructs in these health behaviors, but it is also important to examine the role that these constructs play in dietary behavior change. More recently, Anderson and colleagues found that changes in self-efficacy and self-regulation were associated with changes in fat and fruit and vegetable intake in church members participating in an intervention (19). Similarly, Steptoe and colleagues (20) found that changes in FVC over 12 months were predicted by short-term changes in self-efficacy, benefits (i.e., outcome expectations), knowledge, anticipated regret, and encouragement. Both of these studies involved techniques designed to elicit change in these behaviors, leaving the question of what drives natural changes in dietary behaviors unanswered. Examination of the predictors of natural changes in dietary behaviors over time was the goal of the current study.

Guillaumie and colleagues (21), in a systematic review of FVC, reported that habit, motivations and goals, beliefs about capabilities (i.e., self-efficacy), and knowledge were the most consistent predictors of FVC. However, only 25% of the studies reviewed were longitudinal, and the longest follow-up period was 5 weeks. The present study extends this research by examining these psychosocial determinants over an extended period of time (i.e., 5 months) to determine the long-term associations of social cognitive constructs with changes in dietary behaviors.

Social cognitive theory includes constructs that are both internal to the individual (e.g., self-efficacy, goals) as well as external (e.g., social support, environmental influences). When examining psychosocial predictors of dietary behavior, previous studies have found that both internal and external factors are related to behavior. However, evidence suggests that the internal factors are stronger predictors of dietary behavior than external factors (22). As such, we chose to focus our investigation on the internal social cognitive constructs of self-efficacy, outcome expectations, and goals.

The purpose of this study was to examine the utility of social cognitive constructs for explaining natural changes in dietary behaviors in a sample of working adults. We hypothesized that changes in the social cognitive constructs (i.e., self-efficacy, outcome expectations, and nutrition goals) would be positively associated with changes in dietary behaviors (i.e., FVC and low fat food intake). Specifically, we expected self-efficacy to be positively related to FVC and negatively related to low fat food consumption (LFC) (i.e., increase in self-efficacy would be related to decrease in LFC). We expected the same relationships to hold for outcome expectations and goals. Further, we expected self-efficacy to be indirectly related to dietary behaviors through its positive effects on outcome expectations and goals. We also expected outcome expectations to be indirectly related to behavior through its positive effects on goals. That is, we expected outcome expectations and goals to mediate the relationship between self-efficacy and dietary behavior. Additionally, we expected goals to mediate the relationship between outcome expectations and behavior.

## Materials and Methods

### Participants and recruitment

Participants in this study were employes of a large Midwestern University in the US who were recruited via electronic newsletters, flyers, and personal communication. Recruitment materials informed individuals that they would be asked to complete a paper and pencil questionnaire regarding their dietary behaviors and their thoughts about these behaviors. Participants were also informed that they would be asked to complete these measures twice; once within a few weeks of initial contact, and again 5 months later. The 5-month duration was chosen to avoid data collection during holidays and time during which employes would typically be away from the university setting. Interested individuals had to be a university employe and at least 18 years of age to participate. The study was approved by the local Institutional Review Board.

### Measures

#### Demographics

Demographic information was collected with a brief questionnaire. Items included age, race/ethnicity, type of position (faculty/staff/other), and department of employment.

#### Rapid eating assessment for patients

To assess participants’ dietary behaviors, we used several subscales of the REAP (23), a 27-item measure for quickly assessing dietary intake. For the purpose of the present study, the fruit and vegetable intake and intake of low fat foods subscales were calculated and used in analysis. Items reflect how often a participant consumes certain types of food during an average week and are scored on a three-point scale with corresponding answers of usually/often, sometimes, or rarely/never. The answers were then averaged to give a total score for each of the subscales used.

#### Weight-efficacy lifestyle questionnaire

Self-efficacy for eating behaviors was assessed using the WEL (24). This 20-item measure was designed to evaluate an individual’s self-efficacy for avoiding eating in certain situations. Examples of items include “I can resist eating when I am anxious” and “I can control my eating on the weekends.” Participants then rated their level of confidence in their ability to not eat food during the provided situations on a 10-point scale, ranging from 0 or not confident to 9, very confident. The total WEL scale was then calculated using the mean score of the 20 items.

#### Outcome expectations for nutrition

Outcome expectations for nutrition behaviors were assessed using the 20-item OEN (25). Items include statements reflecting the beliefs that consuming the proper amounts of fruits and vegetables (10 items) or low fat foods (10 items) will bring about certain consequences, both positive and negative. Example items include “If I ate five servings of fruits and vegetables every day I would have more energy” and “If I ate foods low in fat every day I would have to give up all my favorite foods.” Participants were asked to rate their agreement on a five-point Likert scale from “strongly agree” to “strongly disagree.” Mean scores were calculated for each subscale.

#### Nutrition goal-setting scale

To assess nutritional goal-setting, we modified the measure used by Rovniak and colleagues (26). Examples of such items are “I often set nutrition goals” and “I have developed a series of steps for reaching my nutrition goals.” The responses were scored on a five-point scale from 1 (“does not describe”) to 5 (“describes completely”). To obtain the total score for nutrition goal-setting, the mean score of the items was calculated. Mean values and internal consistencies for all measures can be found in Table 1.

**Table 1:** **Descriptive statistics for social cognitive and nutrition outcome variables (*n* = 179)**.

	Baseline			Follow-up			Change	
	*M*	SD	α	*M*	SD	α	F	*p*
Fruit and vegetable consumption	2.1	0.6	0.63	2.0	0.7	0.72	0.11	0.75
Low fat food consumption	2.2	0.4	0.84	2.2	0.4	0.85	0.13	0.72
Self-efficacy	6.3	1.5	0.94	6.2	1.5	0.94	0.21	0.65
Outcome expectations for fruit and vegetable	4.1	0.5	0.70	4.0	0.6	0.70	1.15	0.29
Outcome expectations for low fat foods	3.9	0.6	0.82	3.8	0.6	0.81	1.57	0.21
Nutrition goals	2.3	1.0	0.93	2.2	1.0	0.92	2.40	0.12

### Procedures

#### Data collection

All participants were mailed a packet of questionnaires, an informed consent form, and a stamped, addressed return envelope. Participants were instructed to complete all materials and then return them in the envelope provided. If packets were not returned within 4 weeks, participants were sent a reminder e-mail to encourage them to complete their data and return their packets. Participants who still did not return their packets received another e-mail reminder and a phone call until it had reached 2 months and they were considered lost to follow-up. Participants completed all measures again, 5 months after the baseline collection.

#### Data analysis

**Figure 1:**
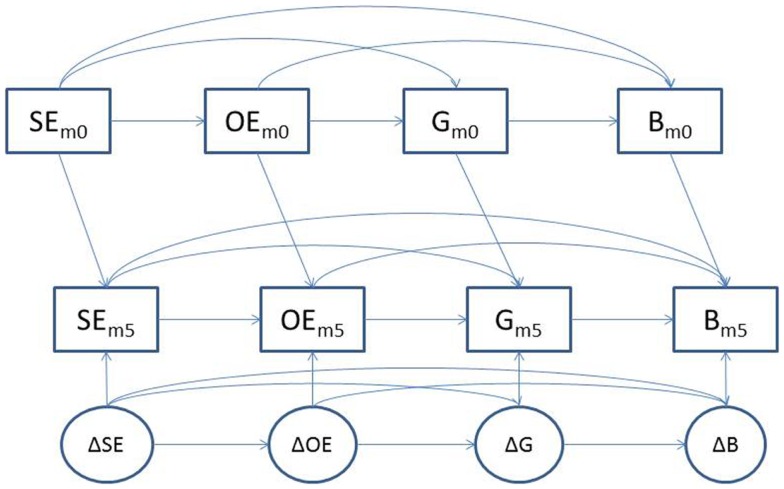
**Panel analysis model showing parallel relationships between social cognitive constructs and nutrition behavior at baseline and month 5 and residualized change (disturbance terms) for each variable**. Note: SE, self-efficacy; OE, outcome expectations, G, goals, B, behavior (either FVC or LFC).

To determine initial associations among the social cognitive variables and dietary outcomes at both time points, Pearson product–moment correlations were estimated using SPSS (v. 20.0). The longitudinal associations of the social cognitive constructs with dietary behaviors were examined using a standard covariance modeling framework to test a modified panel model (see Figure 1). In this model, each month 5 variable was predicted by its corresponding baseline score and disturbances were estimated to index the amount of residualized change in each variable. Those residualized change scores (i.e., disturbances) were permitted to covary to test our hypotheses about changes in one variable predicting changes in another (e.g., changes in self-efficacy predicting changes in outcome expectancies). Within each measurement occasion, covariances between constructs were specified as outlined in SCT. Specifically, (a) behavior was permitted to covary with goals, outcome expectancies, and self-efficacy, (b) goals were permitted to covary with both outcome expectancies and self-efficacy, and (c) outcome expectancies were permitted to covary with self-efficacy. Covariances between residualized change scores also were specified in this manner to test our hypotheses. This model specification isolates change in each variable from baseline to month 5 by controlling for both baseline levels of a construct and concurrent month 5 social cognitive influences on that construct. We conducted two panel analyses, one for FVC and one for LFC as the outcome variables. The model also controlled for the association of BMI, age, and gender with each of the social cognitive and behavioral variables (these paths are not shown in Figure 1 for presentational clarity).

To account for missing data from those participants who did not respond at both data collection time points, we used the full information maximum likelihood (FIML) estimator with AMOS (v.20.0). To determine the appropriateness of using the FIML estimator, missing data were examined. For baseline data, all variables had between 98.9–100% of data points, suggesting that there were minimal problems with missing data. Missing data due to participant attrition at month 5 follow-up was 23.2%. Thus, the criterion of <25% missing data (27, 28) was met for this data set at both time points. Correlation analyses were conducted to determine if any pattern existed for the missing data. There were no significant correlations between the initial value and whether that variable was missing at follow-up, therefore, we concluded that data were missing at random, which justified the use of the FIML estimator.

## Results

### Participant characteristics

Power analysis was conducted to determine the sample size. To be able to detect a correlation of 0.30 at 90% power, we required a sample of 109. A total of 284 employes expressed initial interest in the study with 204 being enrolled in the study after meeting inclusionary criteria. Individuals who declined to participate in the study were either unable to commit to the duration (*n* = 7), no longer interested after they received a full description of the study (*n* = 14), were relocating (*n* = 5), were no longer employed with the university (*n* = 7), had a physical condition that limited their activity (*n* = 2), or failed to contact us after they were sent further details, and contacted several times (*n* = 45). Of the 204 packets originally sent out, 179 were returned completed (87.8% return rate). Characteristics of the sample can be seen in Table 2. Briefly, participants were mostly female (79.3%), White (87.6%), and middle aged (*M* = 45.5 years). There were 137 (77.4%) participants categorized as staff, 16 (9.0%) as faculty, and 24 (13.6%) who identified themselves as other, including academic professionals.

**Table 2:** **Baseline demographic characteristics of sample (*n* = 179)**.

Variable	*n*	(%)	*M*	SD	Range
Age			45.5	11.4	20–70
Sex
Female	142	79.3			
Male	37	20.7			
Race
White	156	87.6			
Black	13	7.3			
Asian	5	2.8			
Latino/a	2	1.1			
Native American	1	0.6			
Other	1	0.6			
BMI			26.41	5.01	18.02–46.30

Of the 179 participants who completed the baseline data collection, 144 (80.5%) provided follow-up data 5 months later. Thirty-five participants failed to complete the follow-up data collection for a variety of reasons including relocation (*n* = 4), pregnancy (*n* = 2), injury/illness (*n* = 2), and failure to return materials (*n* = 27). There were no significant differences on the social cognitive and behavioral outcome variables at baseline between completers and drop-outs; however, those who dropped out tended to be younger [*F*(1, 178) = 4.19, *p* < 0.05], non-White [*F*(1, 178) = 16.04, *p* < 0.01], and male [*F*(1, 178) = 4.02, *p* < 0.05].

### Correlation analysis

Correlation coefficients are provided in Table 3. The majority of the constructs were significantly related with a few exceptions. Specifically, WEL scores (both baseline and follow-up) were unrelated to outcome expectations for either fruit and vegetable or LFC and to FVC (as measured by the REAP). At follow-up, the WEL was found to be unrelated to LFC. However, all other constructs were significantly related at both baseline and follow-up time points.

**Table 3:** **Bivariate correlations among all measured variables at baseline and follow-up**.

	*1*	*2*	*3*	*4*	*5*	*6*	7	8	9	10	11	12
*1. Outcome expectations (fruit and vegetable consumption)*	–											
*2. Outcome expectations (low fat food consumption)*	0.57**	–										
*3. Nutrition goals*	0.30**	0.19*	–									
*4. Self-efficacy*	0.08	0.06	0.15*	–								
*5. Fruit and vegetable consumption*	0.30**	0.11	0.25**	0.06	–							
*6. Low fat food consumption*	0.25**	0.39**	0.41**	0.28**	0.28**	–						
7. Outcome expectations (fruit and vegetable consumption)	0.61**	0.49**	0.25**	0.03	0.29**	0.24**	–					
8. Outcome expectations (low fat food consumption)	0.45**	0.71**	0.20*	0.02	0.17*	0.38**	0.69**	–				
9. Nutrition goals	0.16	0.17*	0.53**	0.13	0.26**	0.33**	0.28**	0.27**	–			
10. Self-efficacy	0.08	0.05	−0.07	0.75**	0.10	0.11	0.03	0.05	0.19*	–		
11. Fruit and vegetable consumption	0.20*	0.14	0.23**	0.07	0.68**	0.21*	0.34**	0.18*	0.32**	0.16	–	
12. Low fat food consumption	0.30*	0.39**	0.39**	0.17*	0.32**	0.84**	0.30**	0.39**	0.37**	0.14	0.29**	–

*Note: italicized text denotes baseline, whereas regular text is follow-up*.

*p < 0.05, **p < 0.01.

### Social cognitive determinants of change in dietary behaviors over time

#### Fruit and vegetable consumption

**Figure 2:**
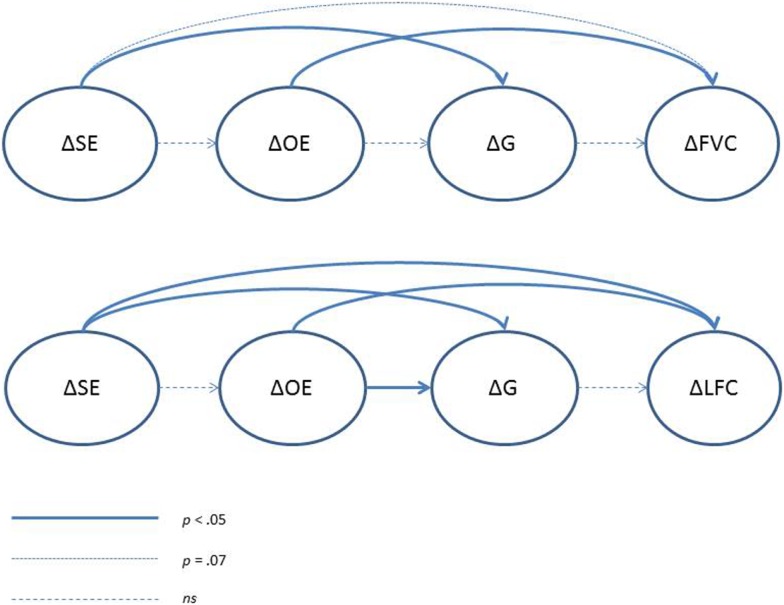
**Diagram of social cognitive determinants of change in dietary behavior over time**. SE, self-efficacy; OE, outcome expectations; G, goals; FVC, fruit and vegetable consumption; LFC, low fat food consumption.

Results for the panel model analyses can be found in Table 4 and visual representation of significant pathways can be found in Figure 2. Overall, the data provided a good fit to the hypothesized model (χ^2^ = 47.78, CFI = 0.95, IFI = 0.96). Changes in outcome expectancies were positively associated with changes in FVC, whereas the association between changes in efficacy beliefs and changes in FVC approached significance (*p* = 0.07). Changes in goals were not associated with residualized change in FVC. Changes in self-efficacy also predicted changes in goals; however, as the path between goals and FVC was non-significant, this indirect path to behavior was not significant. No demographic variables were associated with the social cognitive variables in this model.

**Table 4:** **Path analysis results predicting change in fruit and vegetable and low fat food consumption over time**.

Nutrition outcome	Variable/path	Estimate	SE	*p*
Low fat food consumption	ΔSelf-efficacy → Δnutrition goals	0.18	0.07	0.01
	ΔOutcome expectations (low fat) → Δnutrition goals	0.31	0.17	0.07
	ΔSelf-efficacy → Δoutcome expectations (low fat)	−0.04	0.04	0.36
	m0 Low fat food consumption → m5 low fat food consumption	0.84	0.03	0.00
	ΔSelf-efficacy → m5 low fat food consumption	0.04	0.02	0.02
	ΔOutcome expectations (low fat) → m5 low fat food consumption	0.09	0.04	0.02
	ΔNutrition goals → m5 low fat food consumption	0.00	0.02	0.87
Fruit and vegetable consumption	ΔSelf-efficacy → Δnutrition goals	0.18	0.07	0.01
	ΔOutcome expectations (fruit and vegetable) → Δnutrition goals	0.21	0.19	0.27
	ΔSelf-efficacy → Δoutcome expectations (fruit and vegetable)	−0.04	0.04	0.38
	m0 Fruit and vegetable consumption → m5 fruit and vegetable consumption	0.75	0.05	0.00
	ΔSelf-efficacy → m5 fruit and vegetable consumption	0.07	0.04	0.07
	ΔOutcome expectations (fruit and vegetable) → m5 fruit and vegetable consumption	0.28	0.08	0.00
	ΔNutrition goals → m5 fruit and vegetable consumption	0.06	0.05	0.18

#### Low fat food consumption

The data provided a reasonable fit to the hypothesized model fit, although it could be improved upon (χ^2^ = 88.01, CFI = 0.91, IFI = 0.92). Significant paths were found between changes in outcome expectations and changes in LFC as well as changes in self-efficacy and changes in LFC. Changes in goals were predicted by both changes in outcome expectations and changes in self-efficacy. However, the path from changes in goals to changes in LFC was non-significant. Participants with higher BMI tended to consume more high fat foods than those with lower BMIs.

## Discussion

In the present study, we examined the role of several social cognitive factors in changes in dietary behaviors (i.e., FVC and LFC) in university employes over the course of 5 months. We found differences for FVC and LFC in terms of predictors of change with only outcome expectations being positively directly associated with change in FVC, although self-efficacy trended in the expected direction. Both outcome expectations and self-efficacy were positively directly associated with change in LFC over 5 months. Counter to our hypotheses, goals were not associated with change in either behavior. Additionally, there were no significant indirect pathways to changes in behavior (i.e., there were no significant mediation pathways for either behavior).

Outcome expectations emerged as a positive significant independent predictor of change in both FVC and LFC, a finding that is consistent with SCT and other reports. For example, Anderson et al. (18) reported that negative outcome expectations were related to fat and fruits and vegetable levels, though this effect was partially indirect through self-regulatory strategies. Further, Steptoe et al. (20) found that increases in FVC were predicted by increases in perceived benefits (as well as self-efficacy). Interestingly, Van Duyn and colleagues found that perceived benefits were associated with increased FVC in males only (22). As the present sample was primarily comprised of women, it would appear that outcome expectations may be important for both males and females, although we would hesitate to make strong conclusions regarding the possible moderating role that gender plays in the relation between outcome expectations and dietary behaviors.

Self-efficacy was found to be a modest, but statistically significant predictor of change in consumption of low fat foods, but only trended in that direction for fruits and vegetables. This finding is supported by research by Smith Anderson-Bill et al. (29), who also found that self-efficacy was significantly related to fat consumption, but not to fruit and vegetable intake. However, typically speaking, self-efficacy is a major contributor to behavior change (30, 31). That our findings were less robust may be explained by the measure of self-efficacy, which focuses on resistance eating in tempting situations. For example, such situations in which one might be tempted by foods that are high in fat content might include social engagements and reactions to emotional events. Thus, a social cognitive perspective would take the view that resisting LFC is a more challenging eating behavior than consuming fruit and vegetables.

Although goal-setting was significantly related to both FVC and LFC at baseline, it was not found to be a significant predictor of change in healthy eating practices in this study, contrary to other studies. For example, although McEachan et al. reported intentions (or goals) to be better predictors of physical activity than nutrition behaviors (32), Kalavana and colleagues found that goal efficacy and ownership were associated with healthy eating behaviors in adolescents (33). It could be that the act of setting a goal is not enough to bring about actual behavior change. There is a documented gap between intentions and behavior in the literature (34). Turning these goals or intentions into actual behavior may take more self-monitoring or self-regulatory processes such as planning (31). Changes in planning were found to be associated with changes in FVC in a longitudinal study (35) and an implementation intentions intervention was found to be effective at improving FVC in people with high levels of self-efficacy (36). Further exploration into the role of goal-setting in dietary behavior change is warranted. It may be more important to determine the implementation strategies for that goal than simply setting the goal itself.

We tested both the direct and indirect effects of self-efficacy and outcome expectations on dietary behaviors. Although pathways between changes in self-efficacy, outcome expectations, and goals were significant, the indirect path to either behavior was not significant (i.e., from goals to the behavior). Perhaps goal-setting practices do not exhibit natural fluctuations in the same way as self-efficacy and outcome expectations. This indirect pathway may be found to be significant during a goal-setting and implementation intervention where participants learn to use effective goal-monitoring strategies.

Findings suggest that interventions to improve dietary behaviors may be most effective if they focus on modification of self-efficacy and outcome expectations. Improving a person’s confidence in their ability to eat in a healthy way may result in behavior change. Further, educating employes on the beneficial outcomes associated with healthy eating may also result in behavior change within a worksite setting. Currently, more than 154 million Americans comprise the national civilian workforce (37). Worksites are a setting at which individuals from a variety of life-stages and of varying socio-economic status can be reached in an efficient manner. Thus, structuring behavior change interventions based on theoretical findings such as these may lead to positive health outcomes in a wide audience. These results show which theoretical constructs should be targeted to have the potential to improve behavior. However, these implications should be taken with some caution.

### Limitations and future directions

Although there are several strengths to the present study (i.e., relatively long duration, exploration of change in behavior), it is not without limitation. First, the study would have benefited from context-specific measures of social cognitive constructs. Emerging research in the physical activity literature (38) called the scoring structure of the goals measure into question. Development of a measure of goal implementation and planning is also needed for nutritional behaviors. Second, the size of the sample could have influenced the results. Although the response rate for this sample was relatively high and the retention rate was acceptable, larger-scale studies with greater diversity are warranted. Additionally, there was a systematic drop-out of participants. There were no significant differences in the main variables in our analyses; however, the estimates may be biased and should be interpreted with some caution. It is possible that unmeasured variables such as knowledge of nutrition, and resources for nutrition could have contributed to this pattern of attrition, but this is speculative.

## Summary

In sum, this study was able to demonstrate some of the social cognitive predictors of healthy dietary behaviors in a sample of university employes. Extension of this work should be conducted to better understand the complex nature of dietary behaviors. Promotion of healthy dietary behaviors should include efforts to increase outcome expectations and self-efficacy for eating healthy foods. Additionally, goal-setting strategies may be effective, but this hypothesis requires further testing.

## Conflict of Interest Statement

The authors declare that the research was conducted in the absence of any commercial or financial relationships that could be construed as a potential conflict of interest.
